# Insights from the Dalberto Teixeira Pombo (DTP) Arthropod Collection – II. Long-term monitoring of arthropod fauna in the show cave Algar do Carvão (Terceira, Azores, Portugal)

**DOI:** 10.3897/BDJ.13.e167838

**Published:** 2025-11-19

**Authors:** Luís Carlos Fonseca Crespo, Isabel R. Amorim, Fernando Pereira, Paulo A.V. Borges

**Affiliations:** 1 University of the Azores, CE3C—Centre for Ecology, Evolution and Environmental Changes, Azorean Biodiversity Group, CHANGE —Global Change and Sustainability Institute, School of Agricultural and Environmental Sciences, Rua Capitão João d’Ávila, Pico da Urze, 9700-042, Angra do Heroísmo, Azores, Portugal University of the Azores, CE3C—Centre for Ecology, Evolution and Environmental Changes, Azorean Biodiversity Group, CHANGE —Global Change and Sustainability Institute, School of Agricultural and Environmental Sciences, Rua Capitão João d’Ávila, Pico da Urze, 9700-042 Angra do Heroísmo, Azores Portugal; 2 IUCN SSC Atlantic Islands Invertebrate Specialist Group, Angra do Heroísmo, Azores, Portugal IUCN SSC Atlantic Islands Invertebrate Specialist Group Angra do Heroísmo, Azores Portugal; 3 University of the Azores, CE3C—Centre for Ecology, Evolution and Environmental Changes, Azorean Biodiversity Group, CHANGE —Global Change and Sustainability Institute, Rua Capitão João d’Ávila, Pico da Urze, 9700-042, Angra do Heroísmo, Azores, Portugal University of the Azores, CE3C—Centre for Ecology, Evolution and Environmental Changes, Azorean Biodiversity Group, CHANGE —Global Change and Sustainability Institute, Rua Capitão João d’Ávila, Pico da Urze, 9700-042 Angra do Heroísmo, Azores Portugal; 4 IUCN SSC Monitoring Specialist Group, Angra do Heroísmo, Azores, Portugal IUCN SSC Monitoring Specialist Group Angra do Heroísmo, Azores Portugal

## Abstract

**Background:**

The second manuscript in the series "Dalberto Teixeira Pombo (DTP) Arthropod Collection" focuses on Algar do Carvão, a remarkable volcanic pit on Terceira Island, Azores, that is a Natural Monument, a show cave and part of the Terceira Island Natural Park. This volcanic cave is unique amongst the archipelago’s subterranean systems due to its distinctive geological features, including rare silica-based speleothems and its exceptional natural setting. Surrounded by remnants of native laurel forest, the cave hosts a specialised assemblage of arthropods, including several taxa endemic to the Azores and single island endemic species. Of particular interest are four obligate cave-dwelling species or subspecies (troglobionts): the centipede *Lithobius obscurus azoreae* Eason & Ashmole, 1992 (Chilopoda, Lithobiomorpha, Lithobiidae); the springtail *Pseudosinella ashmoleorum* da Gama, 1988 (Collembola, Entomobryomorpha, Entomobryidae); the spider *Turinyphia cavernicola* Wunderlich, 2008 (Arachnida, Araneae, Linyphiidae); and the ground beetle *Trechus terceiranus* Machado, 1988 (Insecta, Coleoptera, Carabidae), the last two being endemics to Terceira Island. These species are part of a fragile and narrowly distributed subterranean fauna shaped by the volcanic origin of the island and its isolation (0.4 Ma). Their presence highlights the conservation value of Algar do Carvão, which serves not only as a cave biodiversity hotspot, but also as a natural laboratory for studying evolution, adaptation to subterranean habitats and island biogeography. Despite being a show cave with frequent human visits, Algar do Carvão retains a relatively intact hypogean ecosystem, though it remains vulnerable to anthropogenic pressures, such as habitat disturbance and pollution.

**New information:**

Particular focus is given to the abundance and population trends of the endemic cave-adapted beetle *Trechus terceiranus*, monitored through a long-term standardised programme initiated in 1999 using non-lethal trapping methods. This effort, designed to monitor population variation across seasons and years and to evaluate the potential impacts of increasing human visitation to the cave, represents one of the few continuous monitoring programmes for cave arthropods on oceanic islands. Over the course of this study, we also report the first citation of 21 arthropod taxa for Algar do Carvão, two of which are endemic to the Azores (*Canariphantes acoreensis* (Wunderlich, 1992) and *Phloeostiba azorica* (Fauvel, 1900)), contributing significantly to the known biodiversity of this volcanic pit, in particular and of Azorean subterranean fauna, in general.

## Introduction

### Monitoring

Long-term monitoring is a cornerstone of evidence-based conservation, especially for cave-adapted species that exhibit high levels of endemism, limited distributions and extreme sensitivity to environmental change (Mammola et al. 2022). Subterranean ecosystems, including volcanic caves (lava tubes and volcanic pits) and karst systems, host unique biological communities composed largely of obligate troglobionts - species that have evolved specialised traits, such as loss of pigmentation and eyesight and which complete their entire life cycle underground (Gibert and Deharveng 2002).

Monitoring efforts provide critical baseline data on species abundance, distribution and ecological requirements, enabling the detection of population trends, emerging threats and the effectiveness of conservation interventions (Stephenson et al. 2022). This is especially vital on islands, where many troglobiont species occur in only one or two caves and are, thus, extremely vulnerable to stochastic events, habitat degradation and anthropogenic pressures (Borges et al. 2012).

The lack of long-term data has been identified as a major impediment in subterranean conservation, leading to reliance on sporadic data, anecdotal evidence and expert opinion rather than rigorous, quantitative studies (Mammola et al. 2020). Furthermore, threats such as pollution, tourism and climate change, often manifest subtly and cumulatively, requiring continuous observation to understand their impact (Reboleira et al. 2011, Borges et al. 2012, Mammola et al. 2020, Mammola et al. 2022).

Ultimately, long-term monitoring is not merely a scientific necessity, it is an ethical imperative for preserving some of the planet's most ancient and fragile forms of life (Wynne et al. 2021, Stephenson et al. 2022).

### Touristic pressure

The impacts of tourism on show caves can be manifold. These were recently reviewed by Piano et al. (2024), who classified five interlinked groups of impacts: physical changes, chemical changes, pollutants, allochthonous microorganisms and disturbance to subterranean organisms. Those authors also proposed several response types as countermeasures to the referred impacts, namely protection, regulation, restoration and indirect measures.

Several case studies tested the impact of touristic activity on show caves on native invertebrate communities. To cite a few, Pacheco et al. (2020) used quadrat sampling to analyse the effects of tourism in Gruta de Lanquín (Guatemala), showing a change on community structure dependent on the presence of touristic activity. Others, such as Faille et al. (2014), failed to find differences in abundance and species richness evaluators in La Verna Cave (France), showing that a certain degree of resilience can also be found in cave-adapted organisms, especially when inhabiting a large cave.

### Geography

The Azores are a volcanic archipelago composed of nine islands, located in the North Atlantic Ocean roughly between 37─40° N and 25─31° W. These islands have different geological ages, spanning from the 6 Ma of Santa Maria to the 0.19 Ma of Pico (Florencio et al. 2021). As in other volcanic archipelagoes, such as Hawaii or the Canary Islands (Pipan and Culver 2019), subterranean habitats are present in the Azores, generated by the cooling of lava flows and can assume two main types: lava tubes or volcanic pits.

**Figure 1.**
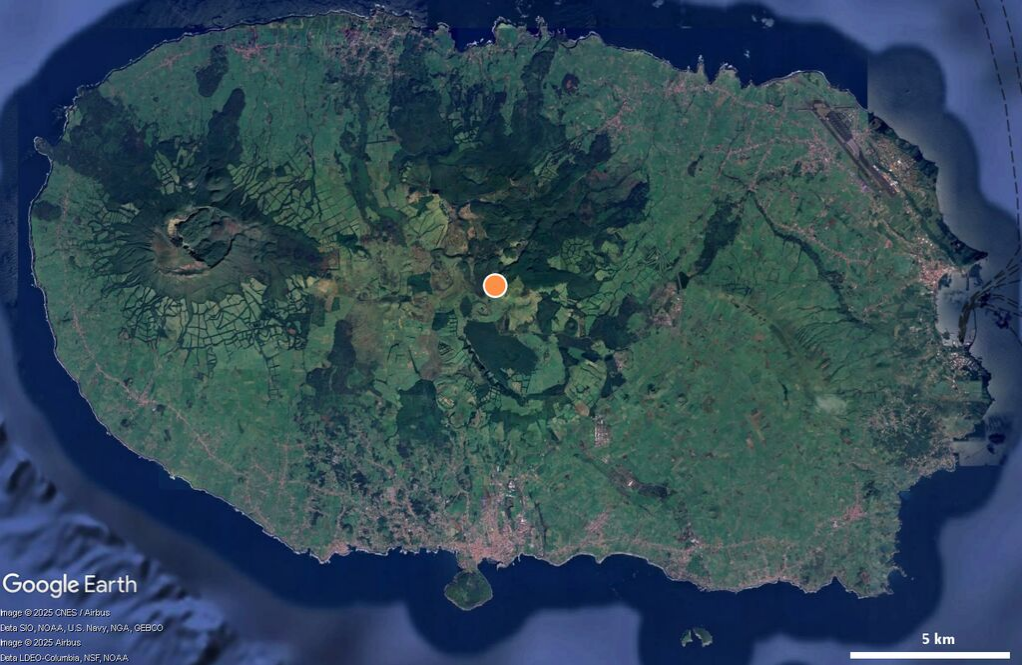
Map of Terceira, Azores. Red dot - location of Algar do Carvão. Image source: Google Earth.

**Figure 2.**
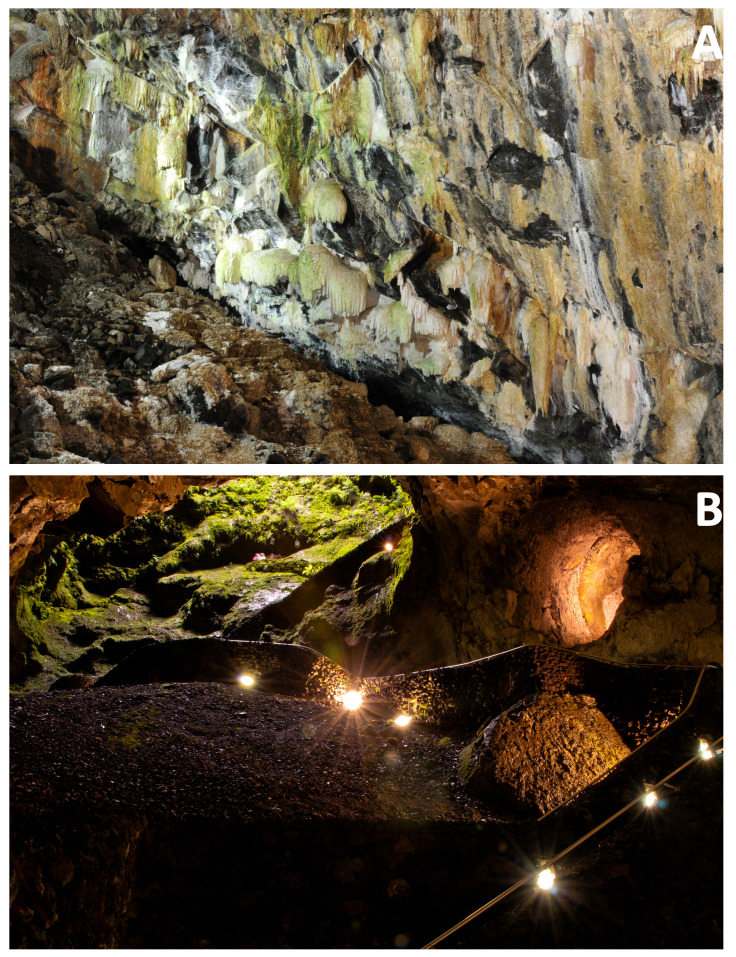
Algar do Carvão. **A** wall of the cave with numerous silica-based speleothems; **B** entrance pathway used by visitors. Photo credits: Paulo A.V. Borges.

**Figure 3.**
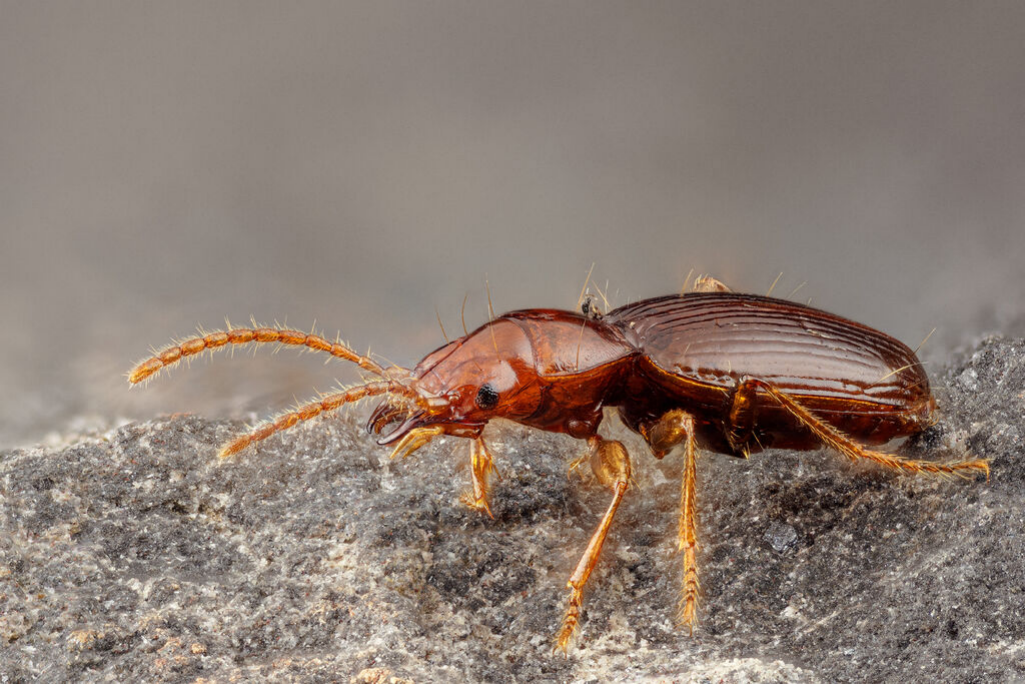
*Trechus terceiranus*. Photo credit: Javier Torrent (Azorean Biodiversity Group, CE3C).

Algar do Carvão is a volcanic pit in the Island of Terceira (Fig. 1) and it is one of two show caves of the Island (Gruta do Natal is a lava tube open to the public). It is widely known for its silica-based speleothems (Daza et al. 2014) (Fig. 2) and its walls near the surface are furnished with native vegetation, thus creating a remarkable geological setting, protected by law as a "Regional Natural Monument" since 2004 (overviewed in Mammola et al. (2024): 6, box 3) and safeguarded by the ENGO “Associação Os Montanheiros”, who currently manage the touristic visitation (Fig. 2). In addition, endemic arthropod species present in the IUCN Red List are known to occur in this cavity, such as the endemic beetle *Trechus terceiranus* Machado, 1988 (Borges 1993; Conservation status: Vulnerable, after Borges and Amorim (2018)) (Fig. 3) or the spider *Turinyphia cavernicola* Wunderlich, 2008 (Conservation status: Endangered, after Borges and Cardoso (2017)).

## General description

### Purpose

In this manuscript, we present the arthropod inventorying data collected in the volcanic pit Algar do Carvão since 1999, mostly by the use of non-lethal baited pitfall trapping. Besides citing first time occurrences of several species, we intend to focus on quantifying the abundance of the troglobiont ground-beetle *Trechus terceiranus* throughout the first decade of 2000 and relate it with the increase of touristic activity, to assess any effect caused by the latter. This will allow for a first analysis of long-term abundance data of a cave-adapted single-island endemic species (SIE) in a period of growing global mobility and touristic pressure.

### Additional information

The initial arthropod survey at Algar do Carvão in 1998 involved the deployment of a rudimentary trap, consisting of a plastic cup placed adjacent to a rabbit carcass on the entrance staircase, with vinegar employed as an attractant. Subsequently, three to four pitfall traps were set up with the objective of detecting the presence of *Trechus* beetles. However, it should be noted that the resulting data from these preliminary sampling efforts were not systematically recorded.

**Figure 4.**
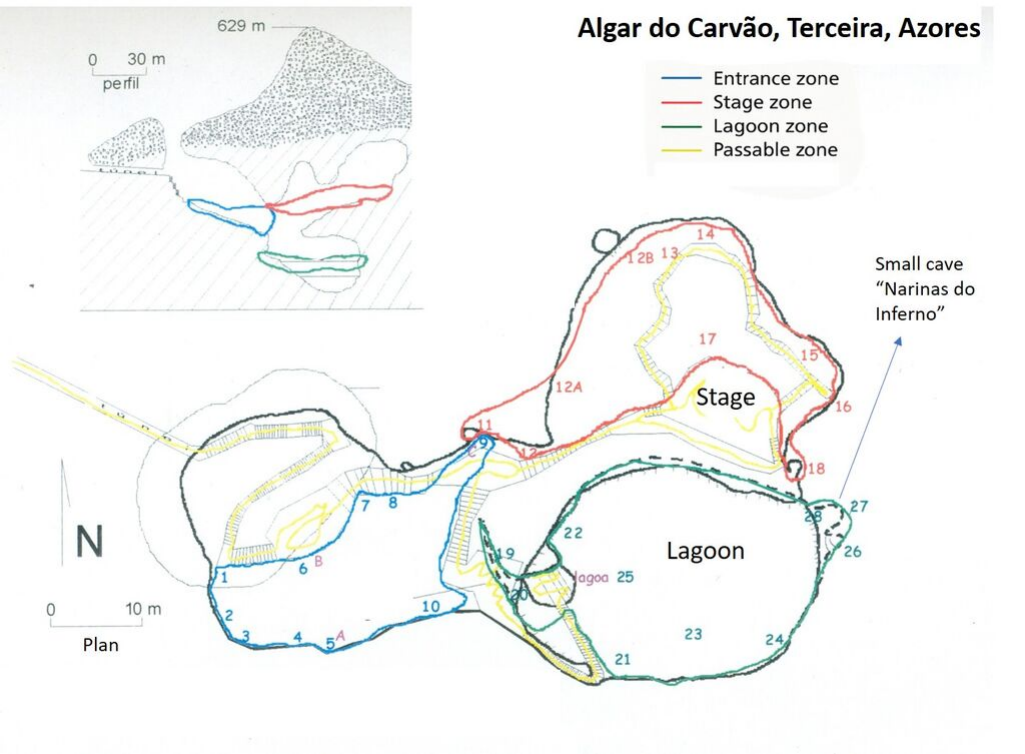
The topography of Algar do Carvão performed by "Os Montanheiros" initially in 1964 and with modifications in 1968 and later in 2003. The plan was made in 2007. The location of the traps is indicated with numbers in the three meain sampled areas: entrance (blue), lagoon (green) and stage (red).

In April 1999, as part of Isabel R. Amorim's doctoral research (Amorim 2005), ten non-lethal pitfall traps were deployed in the entrance area of the cave. Captured *Trechus* beetles were individually marked with small dots of model paint for mark–recapture studies and monitor *Trechus* movements within the cave; microclimatic conditions were also assessed, with temperature and humidity measured. From 1999 to 2001, up to 20 non-lethal pitfalls were set up around the cave (mainly at the entrance - blue area and around the laggon - red area, Fig. 4) at each sampling event to collect live specimens for DNA analyses (Amorim 2005). Non-lethal pitfall trapping was continued from 2002 onwards. The trapping protocol was expanded to include the other sectors of the cave (Fig. 4), maintaining an equal number of pitfall traps in each area of Algar do Carvão. Environmental monitoring was also continued, with regular measurements of air temperature and humidity and, when possible, lagoon water level and temperature.

## Project description

### Title

Inventory and monitoring of a protected species in a show cave of Terceira Island, Azores, Portugal

### Personnel

The original project was conceived by Fernando Pereira, Isabel R. Amorim and Paulo A.V. Borges. The current project was conceived and is being led by Paulo A.V. Borges.

Fieldwork (site selection and experimental setting): Paulo A.V. Borges, Isabel R. Amorim and Fernando Pereira.

Fieldwork (authorisation): Azorean Regional Directorate for the Environment.

Parataxonomists (Laboratory): Fernando Pereira, Isabel R. Amorim and Luís Carlos Crespo.

Taxonomists: Luís Carlos Crespo and Paulo A.V. Borges.

Arthropod Curation: Fernando Pereira, Paulo A.V. Borges and Luís Carlos Crespo.

Darwin Core Databases: Luís Carlos Crespo and Paulo A.V. Borges.

### Study area description

This study was undertaken in a single volcanic pit in Terceira Island, Algar do Carvão (Fig. 2). A current touristic hotspot of the Island, it is located near an area with native vegetation, naturally occurring there, but also restored, in the course of ecosystem recovery processes, following exotic tree plantation in the past. The pit consists of an entrance cavity furnished with native vegetation and, roughly mid-way through the entrance, the human-made hallway is located, descending to greater depths (Pereira et al. 2015). Lateral walls below feature abundant silica-based speleothems and, deep down, a small pond is present. In between the several man-made corridors for the transit of humans, rocky mounds are present. The topography of the cave (Fig. 4) shows the details described above (Pereira et al. 2015).

### Funding

This research was funded by Biodiversa+ (project ‘DarCo’), the European Biodiversity Partnership under the 2021–2022 BiodivProtect joint call for research proposals, co-funded by the European Commission (GA N°101052342) and Fundo Regional para a Ciência e Tecnologia (Portugal). IAR was funded by national funds through FCT – Fundação para a Ciência e a Tecnologia, I.P., under the Norma Transitória https://doi.org/10.54499/DL57/2016/CP1375/CT0003.

Data availability for the general public is funded by the AZORES BIOPORTAL: PORBIOTA (ACORES-01-0145-FEDER-000072).

## Sampling methods

### Sampling description

A single volcanic pit was sampled, with two objectives: a) a faunistic survey of the cave; b) the monitoring of the population of *Trechus terceiranus*.

We used pitfall traps as the most highly effective method for capturing cave-dwelling arthropods, particularly ground beetles of the genus *Trechus*. A small subset of the sampling methods as described in Amorim (2005) was used:

- Trap Setup: Small plastic pitfall traps were placed along the rocky wall of the volcanic pit, from the entrance through the twilight zone to the lake located at the bottom. Traps were dug into the ground and/or placed inside cracks on vertical walls, to maximise the chance of collecting arthropods with distinct lifestyles and covered with mesh to protect against rats. To prevent flooding from dripping water, plastic plates were installed above the traps.
- As Baiting Strategy, one type of baiting was used: Pitfall (bait: liver) - live (non-lethal) traps baited with fresh cow and/or pig liver that was kept in a special container covered with a mesh inside the traps, allowing for the collecton of live specimens; a piece of toilet paper was added, to provide shelter to the live specimens that fell into the traps.
- Pitfall traps were left in place for at least seven days and up to a month to ensure adequate specimen collection. Traps were monitored periodically to prevent potential predation amongst collected specimens, desiccation or disturbance. Occasionally, longer trapping periods occurred due to climatic and/or logistical constraints.

A total of 30 traps were operational, but not all at the same time. The main traps 1-10 located in the main plateau (entrance) of the cave (see Fig. 4 - blue area) were in operation since the beginning of the study (in January 2001). Traps 11 to 18 (including 12, 12A, 12B) were located in the Satge area (Fig. 4 - red area) and its operation started only in 2002. The traps 19 to 28 were located around the lagoon (Fig. 4 - green area) and its operation also started in 2002.

Specimens were counted upon each monitoring and released on the location where they were collected. Some specimens were found dead in the traps and stored in a preservative medium, such as 100% acetone, known to be an optimal preservative for DNA analyses. For logistical reasons, some specimens were initially placed in 70% ethanol (before identification) and subsequently transferred to 100% acetone. Specimens were kept either at -20ºC or at room temperature. After species identification performed for this study, all specimens were stored in 96% ethanol at room temperature. A few sporadic visual direct searches were also conducted in the cave, aiming at a comprehensive faunistic knowledge of Algar do Carvão.

## Geographic coverage

### Description

This study concerns one particular volcanic pit in the Island of Terceira, Algar do Carvão.

**Protection Level of Algar do Carvão, Terceira Island**

Algar do Carvão, located in the central part of Terceira Island in the Azores, is recognised as one of the most significant volcanic caves in the region, both geologically and ecologically. Its protection status has evolved over time, reflecting growing recognition of its natural value.

The initial legal protection for Algar do Carvão was established in 1987, when it was designated as part of the “Geological Natural Reserve of Algar do Carvão and Furna do Enxofre” under Regional Legislative Decree No. 13/87/A of 26 June 1987. This marked the first step in formal conservation efforts for this unique volcanic structure.

In 2004, the area received a higher conservation status, being reclassified as a "Regional Natural Monument" through Regional Legislative Decree No. 9/2004/A of 23 March 2004. This status is reserved for sites of exceptional scientific, cultural or educational value within the Azores and offers enhanced protection measures.

Further integration into the island’s conservation framework occurred in 2011, when Algar do Carvão became part of the Terceira Nature Park, under Regional Legislative Decree No. 11/2011/A of April 2011. This Nature Park aims to ensure the preservation and sustainable management of Terceira’s natural heritage.

Algar do Carvão is also subject to multiple layers of international and regional protection. The area is included within the Serra de Santa Bárbara e Pico Alto Special Area of Conservation (SAC) and is part of the Natura 2000 network, which is the main tool for biodiversity conservation in the European Union. In addition, it is listed as part of the Planalto Central da Terceira Ramsar Site, acknowledging its importance as a wetland of international significance under the Ramsar Convention. Furthermore, Algar do Carvão is recognised as a geosite of the Azores UNESCO Global Geopark, highlighting its geological heritage at the international level and promoting sustainable geotourism (UNESCO Global Geoparks).

Through these designations (regional, national, European and international), Algar do Carvão benefits from a comprehensive framework of protection aimed at preserving its outstanding geological features, endemic biodiversity and ecological functions for future generations.

### Coordinates

38.726 and 38.734 Latitude; -27.218 and -27.205 Longitude.

## Taxonomic coverage

### Description

Phylum: Arthropoda

Class: Arachnida, Insecta, Diplopoda, Chilopoda

Order: Araneae, Chordeumatida, Coleoptera, Dermaptera, Geophilomorpha, Hemiptera, Julida, Lithobiomorpha, Opiliones, Polydesmida, Pseudoscorpiones

Family: Anisolabididae, Blaniulidae, Carabidae, Chthoniidae, Cryptophagidae, Dryopidae, Geophilidae, Haploblainosomatidae, Hydrophilidae, Julidae, Leiobunidae, Leiodidae, Linyphiidae, Lithobiidae, Lygaeidae, Polydesmidae, Staphylinidae, Theridiidae

Due to the lack of specialists for some Arthropod groups, a number of specimens still remain unidentified and are stored in the Dalberto Teixeira Pombo collection - namely, Isopoda, Amphipoda, Acari, Collembola, Diptera, Hymenoptera and Lepidoptera.

## Temporal coverage

### Notes

18-02-1999 to 21-09-2023

## Collection data

### Collection name

Dalberto Teixeira Pombo /NCBI- BioCollections - UAC<PRT>:DTP

### Collection identifier

DTP

### Specimen preservation method

Ethanol 96%

## Usage licence

### Usage licence

Creative Commons Public Domain Waiver (CC-Zero)

## Data resources

### Data package title

Inventory and monitoring of a protected species in a show cave of Terceira Island - Algar do Carvão

### Resource link

https://doi.org/10.15468/5aj4ae

### Alternative identifiers

https://www.gbif.org/dataset/df5c3813-841e-4ac0-96b3-2f54252da02b

### Number of data sets

2

### Data set 1.

#### Data set name

Event table

#### Data format

Darwin Core Archive format

#### Character set

UTF-8

#### Download URL

http://ipt.gbif.pt/ipt/resource?r=cave_trechus_alga

#### Data format version

1.1

#### Description

The dataset was published in the Global Biodiversity Information Facility platform, GBIF (Borges et al. 2025). The following data-table includes all the records for which a taxonomic identification of the specimens was possible. The dataset submitted to GBIF is structured as a sample event dataset that has been published as a Darwin Core Archive (DwCA), which is a standardised format for sharing biodiversity data as a set of one or more data tables. The core data file contains 1370 records (eventID). This GBIF IPT (Integrated Publishing Toolkit, Version 2.5.6) archives the data and, thus, serves as the data repository. The data and resource metadata are available for download in the Portuguese GBIF Portal IPT (Borges et al. 2025).

**Data set 1.** 

Column label	Column description
eventID	An identifier for the set of information associated with a dwc:Event (something that occurs at a place and time). May be a global unique identifier or an identifier specific to the dataset.
samplingProtocol	The names of, references to or descriptions of the methods or protocols used during a dwc:Event.
sampleSizeValue	A numeric value for a measurement of the size (time duration, length, area or volume) of a sample in a sampling dwc:Event.
sampleSizeUnit	The unit of measurement of the size (time duration, length, area or volume) of a sample in a sampling dwc:Event.
samplingEffort	The amount of effort expended during a dwc:Event.
eventDate	The nature of the dwc:Event.
year	The four-digit year in which the dwc:Event occurred, according to the Common Era Calendar.
month	The integer month in which the dwc:Event occurred.
day	The integer day of the month on which the dwc:Event occurred.
habitat	A category or description of the habitat in which the dwc:Event occurred.
fieldNumber	The date-time or interval during which a dwc:Event occurred. For occurrences, this is the date-time when the dwc:Event was recorded. Not suitable for a time in a geological context.
locationRemarks	Comments or notes about the dcterms:Location.
locality	The specific description of the place.
municipality	The full, unabbreviated name of the next smaller administrative region than county (city, municipality etc.) in which the dcterms:Location occurs. Do not use this term for a nearby named place that does not contain the actual dcterms:Location.
dynamicProperties	A list of additional measurements, facts, characteristics or assertions about the record. Meant to provide a mechanism for structured content. In the specific case of the present manuscript, we indicate length or depth of the cave, depending if it is a lava tube or a volcanic pit.
islandGroup	The name of the island group in which the dcterms:Location occurs.
island	The name of the island on or near which the dcterms:Location occurs.
country	The name of the country or major administrative unit in which the dcterms:Location occurs.
countryCode	The standard code for the country in which the dcterms:Location occurs.
stateProvince	The name of the next smaller administrative region than country (state, province, canton, department, region etc.) in which the dcterms:Location occurs.
minimumElevationInMetres	The lower limit of the range of elevation (altitude, usually above sea level), in metres.
decimalLatitude	The geographic latitude (in decimal degrees, using the spatial reference system given in dwc:geodeticDatum) of the geographic centre of a dcterms:Location. Positive values are north of the Equator, negative values are south of it. Legal values lie between -90 and 90, inclusive.
decimalLongitude	The geographic longitude (in decimal degrees, using the spatial reference system given in dwc:geodeticDatum) of the geographic centre of a dcterms:Location. Positive values are east of the Greenwich Meridian, negative values are west of it. Legal values lie between -180 and 180, inclusive.
geodeticDatum	The ellipsoid, geodetic datum or spatial reference system (SRS) upon which the geographic coordinates given in dwc:decimalLatitude and dwc:decimalLongitude are based.
coordinateUncertaintyInMetres	The horizontal distance (in metres) from the given dwc:decimalLatitude and dwc:decimalLongitude describing the smallest circle containing the whole of the dcterms:Location. Leave the value empty if the uncertainty is unknown, cannot be estimated or is not applicable (because there are no coordinates). Zero is not a valid value for this term.
coordinatePrecision	A decimal representation of the precision of the coordinates given in the dwc:decimalLatitude and dwc:decimalLongitude.
georeferenceSources	A list (concatenated and separated) of maps, gazetteers or other resources used to georeference the dcterms:Location, described specifically enough to allow anyone in the future to use the same resources.

### Data set 2.

#### Data set name

Occurrence table

#### Data format

Darwin Core Archive format

#### Character set

UTF-8

#### Download URL

http://ipt.gbif.pt/ipt/resource?r=cave_trechus_algar

#### Data format version

1.1

#### Description

The dataset was published in the Global Biodiversity Information Facility platform, GBIF (Borges et al. 2025). The following data table includes all the records for which a taxonomic identification of the species was possible. The dataset submitted to GBIF is structured as an occurrence table that has been published as a Darwin Core Archive (DwCA), which is a standardised format for sharing biodiversity data as a set of one or more data tables. The core data file contains 1829 records (occurrenceID). This GBIF IPT (Integrated Publishing Toolkit, Version 2.5.6) archives the data and, thus, serves as the data repository. The data and resource metadata are available for download in the Portuguese GBIF Portal IPT (Borges et al. 2025).

**Data set 2.** 

Column label	Column description
eventID	An identifier for the set of information associated with a dwc:Event (something that occurs at a place and time). May be a global unique identifier or an identifier specific to the dataset.
type	The nature or genre of the resource.
licence	A legal document giving official permission to do something with the resource.
institutionID	An identifier for the institution having custody of the object(s) or information referred to in the record.
collectionID	An identifier for the collection or dataset from which the record was derived.
institutionCode	The name (or acronym) in use by the institution having custody of the object(s) or information referred to in the record.
collectionCode	The name, acronym, coden or initialism identifying the collection or dataset from which the record was derived.
datasetName	The name identifying the dataset from which the record was derived.
basisOfRecord	The specific nature of the data record.
occurrenceID	An identifier for the dwc:Occurrence (as opposed to a particular digital record of the dwc:Occurrence). In the absence of a persistent global unique identifier, construct one from a combination of identifiers in the record that will most closely make the dwc:occurrenceID globally unique.
organismQuantity	A number or enumeration value for the quantity of dwc:Organisms.
organismQuantityType	The type of quantification system used for the quantity of dwc:Organisms.
sex	The sex of the biological individual(s) represented in the dwc:Occurrence.
lifeStage	The age class or life stage of the dwc:Organism(s) at the time when the dwc:Occurrence was recorded.
establishmentMeans	Statement about whether a dwc:Organism has been introduced to a given place and time through the direct or indirect activity of modern humans.
dynamicProperties	A list of additional measurements, facts, characteristics or assertions about the record. Meant to provide a mechanism for structured content. In this case, we add reference to the lifestyle (for troglobionts only) and the IUCN conservation status (for endemic species only).
recordedBy	A list (concatenated and separated) of names of people, groups or organisations responsible for recording the original dwc:Occurrence. The primary collector or observer, especially the one who applies a personal identifier (dwc:recordNumber), should be listed first.
identifiedBy	A list (concatenated and separated) of names of people, groups or organisations who assigned the dwc:Taxon to the subject.
dateIdentified	The date on which the subject was determined as representing the dwc:Taxon.
scientificName	The full scientific name, with authorship and date information if known. When forming part of a dwc:Identification, this should be the name in lowest level taxonomic rank that can be determined. This term should not contain identification qualifications, which should instead be supplied in the dwc:identificationQualifier term.
kingdom	The full scientific name of the kingdom in which the dwc:Taxon is classified.
phylum	The full scientific name of the phylum or division in which the dwc:Taxon is classified.
class	The full scientific name of the class in which the dwc:Taxon is classified.
order	The full scientific name of the order in which the dwc:Taxon is classified.
family	The full scientific name of the family in which the dwc:Taxon is classified.
genus	The full scientific name of the genus in which the dwc:Taxon is classified.
specificEpithet	The name of the first or species epithet of the dwc:scientificName.
infraspecificEpithet	The name of the lowest or terminal infraspecific epithet of the dwc:scientificName, excluding any rank designation.
taxonRank	The taxonomic rank of the most specific name in the dwc:scientificName.
scientificNameAuthorship	The authorship information for the dwc:scientificName formatted according to the conventions of the applicable dwc:nomenclaturalCode.
identificationRemarks	Comments or notes about the dwc:Identification.

## Additional information

We report the first occurrence of 21 species in Algar do Carvão, listed in Table 1.

### Discussion

**Figure 5.**
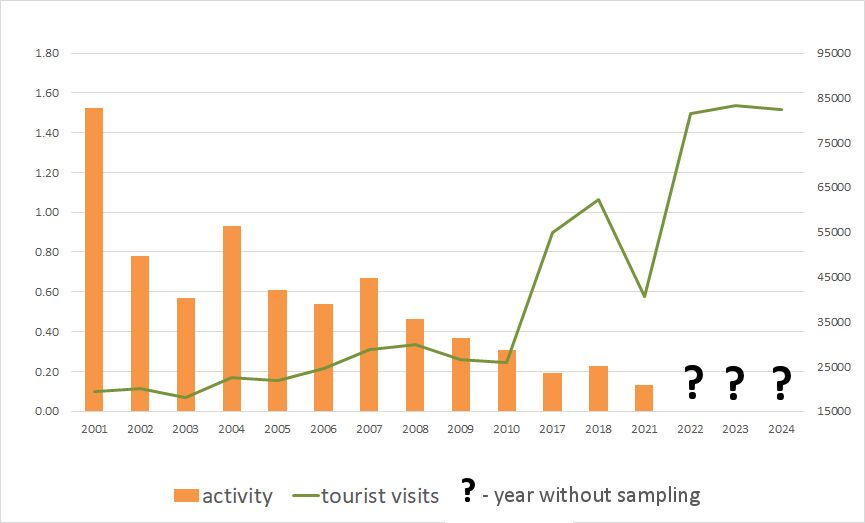
Graphic with the activity / density of *T. terceiranus* in Algar do Carvão (abundance/Nsample/Ndays) and the number of tourists entering the cave (sent by courtesy of Paulo Barcelos / Associação Os Montanheiros). Monitoring data from the periods of 2011-2016 and 2020 are unavailable.

The long-term standardised monitoring of *Trechus terceiranus* in Algar do Carvão revealed a marked decline in the abundance of this endemic troglobiont beetle over a period of intensifying tourist activity in the show cave (Fig. 5). This pattern strongly suggests a negative relationship between human visitation and both the activity and implied viability of subterranean arthropod populations. Similar findings have been documented in other show caves worldwide, where increased foot traffic, artificial lighting, microclimatic alterations and surface-derived pollutants have been shown to negatively impact cave-dwelling fauna, particularly specialised and endemic invertebrates (Reboleira et al. 2011, Wynne et al. 2021, Mammola et al. 2022).

Several mechanisms may explain the decline in *T. terceiranus* abundance. Human presence in caves can lead to increases in temperature, CO₂ levels and humidity fluctuations, which may disrupt the stable microclimatic conditions to which cave-adapted organisms are highly specialised (Mammola et al. 2022). Additionally, the installation of artificial lighting is known to cause phototrophic microbial and plant growth (lampenflora), which can alter food webs and the chemical balance of cave ecosystems and lead to habitat deterioration (Hathaway et al. 2014, Baquedano Estévez et al. 2019, Addesso et al. 2024). The introduction of dust, organic matter and chemical pollutants from tourist clothing and footwear may further contribute to habitat degradation (Wynne et al. 2021).

The year of 2020, with the onset of the Covid-19 pandemic, saw a reduction in the number of visitors to Algar do Carvão, given the implementation of rules of conduct towards crowding situations (16572 visitors, not shown in graphic), but, in 2021, that number more than doubled and was coupled with a low activity of *T. terceiranus*. Moreover, in recent years, there has been a continuous boom of touristic visits, with yearly visitor numbers almost reaching 85000 (Fig. 5). It is paramount to continue monitoring this sensitive cave-adapted endemic species, to contribute to the conservation of this species and avoid risk of local extinction.

To mitigate these impacts, several evidence-based strategies can be implemented. These include limiting the number of visitors and regulating the duration/frequency of visits (carrying capacity management), establishing off-limit zones to protect critical microhabitats and installing less intrusive lighting systems (e.g. motion-activated, low-UV LEDs) (Mammola et al. 2022, Mammola et al. 2024). On a positive note, as a mitigation measure against light pollution in Algar do Carvão, Associação Os Montanheiros (the managing entity) has planned to replace the current lighting fixtures with ones designed to prevent lampenflora growth. Environmental education and visitor awareness programmes are also crucial in promoting responsible behaviour inside caves. Moreover, systematic biodiversity monitoring, as conducted in this study, should be institutionalised as a management tool to guide adaptive conservation actions.

**Figure 6.**
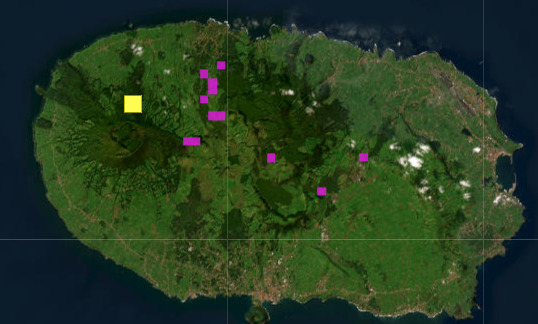
Known distribution for *T. terceiranus*, adapted from the retrieved version downloaded from the Azorean Biodiversity Portal (https://azoresbioportal.uac.pt/pt/especies-dos-acores/trechus-terceiranus-7343/, accessed on 24.IX.2025). Legend: pink squares: cave locations, yellow squares: locations of surveys on the MSS.

The known distribution of *T. terceiranus* is shown in Fig. 6. Here, we can see it only occurs in caves or in the MSS (a survey of this habitat was performed in Borges (1993)). Although a local extinction at Algar do Carvão hardly affects the population of *T. terceiranus* as a whole, given its ability to use neighbouring subterranean habitats, it is paramount to note the effect of touristic pressure on its presence at one of its natural habitats. it is essential that the management strategy at Algar do Carvão better integrates biodiversity conservation objectives with tourism visitation. The adoption of international guidelines, such as those proposed by the IUCN WCPA Working Group on Caves and Karst (Gillieson et al. 2022), could be instrumental in balancing conservation with educational and economic values associated with show caves.

This case study highlights the urgent need to consider subterranean biodiversity in conservation planning and management, particularly in island systems where species often exhibit extreme endemism and narrow ecological tolerances (Wynne et al. 2021, Mammola et al. 2022, Mammola et al. 2024).

## Figures and Tables

**Table 1.** List of species cited for the first time in Algar do Carvão, Terceira, Azores, Portugal. Species in bold are endemic to the Azorean Archipelago.

Class	Order	Family	Species
Arachnida	Araneae	Linyphiidae	***Canariphantes acoreensis* (Wunderlich, 1992)**
			*Palliduphantes schmitzi* (Kulczynski, 1899)
			*Steatoda nobilis* (Thorell, 1875)
	Pseudoescorpiones	Chthoniidae	*Chthonius ischnocheles* (Hermann, 1804)
Chilopoda	Lithobiomorpha	Lithobiidae	*Lithobius pilicornis pilicornis* Newport, 1844
	Geophilomorpha	Geophilidae	*Geophilus truncorum* Bergsøe & Meinert, 1866
Diplopoda	Julida	Blaniulidae	*Nopoiulus kochii* (Gervais, 1847)
		Julidae	*Cylindroiulus latestriatus* (Curtis, 1845)
			*Cylindroiulus propinquus* (Porat, 1870)
			*Ommatoiulus moreleti* (Lucas, 1860)
Insecta	Coleoptera	Carabidae	*Paranchus albipes* (Fabricius, 1796)
		Dryopidae	*Dryops luridus* (Erichson, 1847)
		Hydrophilidae	*Cercyon haemorrhoidalis* (Fabricius, 1775)
		Staphylinidae	*Aleochara verna* Say, 1833
			*Mocyta fungi* (Gravenhorst, 1806)
			*Ocypus aethiops* (Waltl, 1835)
			***Phloeostiba azorica* (Fauvel, 1900)**
			*Proteinus atomarius* Erichson, 1840
			*Trichophya pilicornis* (Gyllenhal, 1810)
	Dermaptera	Anisolabididae	*Euborellia annulipes* (Lucas, 1847)
	Hemiptera	Lygaeidae	*Kleidocerys ericae* (Horvath, 1909)
